# Quantum thermodynamics of single particle systems

**DOI:** 10.1038/s41598-020-70450-y

**Published:** 2020-08-11

**Authors:** Md. Manirul Ali, Wei-Ming Huang, Wei-Min Zhang

**Affiliations:** Department of Physics, National Cheng Kung University, Tainan, 70101 Taiwan

**Keywords:** Quantum physics, Statistical physics, thermodynamics and nonlinear dynamics

## Abstract

Thermodynamics is built with the concept of equilibrium states. However, it is less clear how equilibrium thermodynamics emerges through the dynamics that follows the principle of quantum mechanics. In this paper, we develop a theory of quantum thermodynamics that is applicable for arbitrary small systems, even for single particle systems coupled with a reservoir. We generalize the concept of temperature beyond equilibrium that depends on the detailed dynamics of quantum states. We apply the theory to a cavity system and a two-level system interacting with a reservoir, respectively. The results unravels (1) the emergence of thermodynamics naturally from the exact quantum dynamics in the weak system-reservoir coupling regime without introducing the hypothesis of equilibrium between the system and the reservoir from the beginning; (2) the emergence of thermodynamics in the intermediate system-reservoir coupling regime where the Born-Markovian approximation is broken down; (3) the breakdown of thermodynamics due to the long-time non-Markovian memory effect arisen from the occurrence of localized bound states; (4) the existence of dynamical quantum phase transition characterized by inflationary dynamics associated with negative dynamical temperature. The corresponding dynamical criticality provides a border separating classical and quantum worlds. The inflationary dynamics may also relate to the origin of big bang and universe inflation. And the third law of thermodynamics, allocated in the deep quantum realm, is naturally proved.

## Introduction

In the past decade, many efforts have been devoted to understand how, starting from an isolated quantum system evolving under Hamiltonian dynamics, equilibration and effective thermodynamics emerge at long times^1–5^. On the other hand, the investigations of open quantum systems initiate interests on the issue of quantum thermodynamics taking place under the quantum evolution of open systems^6–20^. The questions of how thermodynamics emerges from quantum dynamics, how do quantum systems dynamically equilibrate and thermalize, and whether thermalization is always reachable in quantum regime, are central and fundamental to research for quantum thermodynamics. However, a general theory of quantum thermodynamics that has conceptually no ambiguity in answering the above questions has not yet been obtained, because investigations in addressing above questions inevitably take various assumptions and approximations. In this paper, we will attempt to answer these questions by rigorously solving the quantum dynamics based on the exact master equation we developed recently for a large class of open quantum systems^21–25^.

Recall that thermodynamics is built with the hypothesis of equilibrium^26^. Macroscopic systems at equilibrium are fully described by the relation between the internal energy *E* and a set of other extensive parameters, the entropy *S*, the volume *V*, and the particle number $N_i$ of different components $i=1, 2, \cdots$, magnetic moment **M**, etc.,

1 $$\begin{aligned} E=E(S, V, N_1, N_2, \cdots , \mathbf{M}, \cdots ), \end{aligned}$$

under the extremum principle of either maximizing the entropy or minimizing the internal energy. The relation of Eq. (1) is called *the fundamental equation* of thermodynamics. The thermodynamical intensive parameters, such as temperature *T*, the pressure *P*, the chemical potential $\mu _1, \mu _2, \cdots$ and magnetic field $\mathbf{B}$, etc. are defined by the first derivative of energy with respect to entropy, the volume and the particle number, etc.

2 $$\begin{aligned} T= \frac{\partial E}{\partial S}, ~P=\frac{\partial E}{\partial V}, ~\mu _i= \frac{\partial E}{\partial N_i}, ~\mathbf{B} = \frac{\partial E}{\partial \mathbf{M}}, \cdots , \end{aligned}$$

such that

3 $$\begin{aligned} dE= TdS + PdV + \sum _i \mu _i dN_i + \mathbf{B} \cdot d\mathbf{M} + \cdots . \end{aligned}$$

These first derivatives in Eq. (2) provide the complete set of equations of state in thermodynamics.

Microscopically (statistical mechanically), all thermodynamic parameters at equilibrium (and therefore the thermodynamic fundamental equation) can be computed from the probability distribution $\{ p_i \}$ over all possible microstates, which can be determined by maximizing Shannon entropy $S=- k \sum _i p_i \ln p_i$ under the condition of fixed average energy (canonical ensemble) or fixed both average energy and average particle number (grand canonical ensembles) in the thermodynamic limit, where the system contains a large number of particles ($\sim 10^{23}$) and couples very weakly to a reservoir. Here we have multiplied the information theoretic Shannon entropy with the Boltzmann constant *k* to make it to be the thermal entropy. The resulting equilibrium probability distribution is given by the equilibrium density matrix

4 $$\begin{aligned} \rho _E = \left\{ \begin{array}{ll} \frac{1}{Z} e^{-\beta H}, &{} \mathrm{canonical~ensemble}; \\ ~&{}~ \\ \frac{1}{Z} e^{-\beta (H-\sum _i \mu _i N_i) }, &{} \mathrm{grand~canonical~ensemble} , \end{array} \right. \end{aligned}$$

where *Z* is the partition function determined by the normalized condition $\mathrm{tr} \rho _E=1$, $\beta =1/kT$ is the inverse temperature of the reservoir, and *H* is the Hamiltonian of the system. Thus, the thermodynamic fundamental equation can be manifested from all the thermodynamic extensive parameters calculated from the thermal equilibrium state $\rho _E$:

5 $$\begin{aligned} \langle A\rangle = {{\,\mathrm{Tr}\,}}[ A \rho _E ] , ~ A= H, N_i, ~\cdots , \mathbf{M},~\cdots ~, ~ \mathrm{and} ~~S = - k_B \mathrm{Tr}[ \rho _E \ln \rho _E] . \end{aligned}$$

In the energy basis, the entropy in Eq. (5) is identical to the Shannon entropy. Equations (1)–(5) provide the axiomatic description of thermodynamics and statistical mechanics, from which all the thermodynamical laws can be obtained naturally, as presented in the classic textbook by Callen^26^.

The left unsolved problem in thermodynamics is the hypothesis of equilibrium itself, i.e. over a sufficiently long time period, a given macroscopic system can always reach thermal equilibrium with its environment (reservoir), and the corresponding equilibrium statistical distribution does not depend on its initial state^27,28^. This process is simply called *thermalization* in the literature. Solving the problem of thermalization within the framework of quantum mechanics is one of the big challenges in physics that has been an old dream for many physicists^29^, and it is also the foundation in the investigation of quantum thermodynamics and non-equilibrium statistical mechanics^30^. No doubt, the solution of thermalization relies on a deep understanding of the dynamics of systems interacting with their environments, i.e. the quantum solution of non-equilibrium time evolution of open systems. In the 19th century, physicists had already developed the classical kinetic theory, or more specifically the Boltzmann’s H-theorem to address the problem^31,32^. Unfortunately, it was not until the 20th century to be realized that microscopic particles in thermodynamical systems, such as atoms, electrons, and photons, followed the rules of quantum mechanics which provides the underlying principle for thermalization.

In fact, Caldeira and Leggett explored the thermalization problem from the principle of quantum mechanics by solving the quantum Brownian motion with the Feynman-Vernon influence functional approach in 1980’s. They recovered both equilibrium statistical mechanics and linear response theory in the asymptotical time limit without taking Markovian approximation^29^. Furthermore, in the beginning of 2000’s, Ford and O’Connell solved exactly the exact master equation of quantum Brownian motion in the Wigner representation derived by Hu, Paz and Zhang^33^ and obtained the asymptotic thermal state from initial Gaussian wavepackets^34^. Zurek explored the nontrivial problem of quantum-to-classical transition and revealed the thermalization as a consequence of entanglement between the system and the reservoir that is realized through decoherence dynamics^35^. In all these investigations, thermalization is demonstrated mainly in quantum Brownian motion with initial Gaussian wave packets at high temperature limit^29,34,35^. In the last decade, we have also derived the exact master equation for a large class of open quantum systems^21–25^ and solved the exact master equation with arbitrary initial states at arbitrary initial temperature of the reservoir such that the thermalization process is generally provided^36–38^. From such exact solutions for a large class of open quantum systems, we will attempt to understand thermodynamics with the detailed thermalization process determined from the principle of quantum mechanics in this paper.

Meanwhile, the aim of quantum thermodynamics is also to develop a thermodynamics formulation far from equilibrium for nanoscale or atomic-scale quantum systems, where the particle number is much less than the order of $10^{23}$. If one can develop quantum thermodynamics for arbitrary small quantum systems, even for single particle quantum systems, and prove the consistency with equilibrium thermodynamics, such quantum thermodynamics should be generally valid for arbitrary dynamics of quantum systems. Therefore, in this paper, we shall focus on the quantum thermodynamics far from equilibrium for *single particle systems coupled strongly or weakly to the reservoir at arbitrary temperature, including the zero-temperature at the deep quantum realm*. Quantum mechanically, a closed system in a pure state remains always in pure states so that no concept of thermalization can be addressed unless the system is quenched^5^. However, when the system is (even for a single particle system) coupled with a reservoir, the system must be entangled with the reservoir so that the system can evolve into mixed states. Thus, entropy is generated and thermalization naturally emerges even for single particle systems coupled strongly or weakly to reservoirs. Hence, we will start from the full quantum dynamics evolution of the system in contact to reservoirs. We investigate the emerging thermodynamics through the non-equilibrium quantum evolution of thermodynamic parameters far from equilibrium, i.e., energy and entropy etc., much before the system approaches to the steady state. More specifically, we shall build quantum thermodynamics completely with the first principle of quantum mechanics.

## Results

### Formulation of quantum thermodynamics far from equilibrium

**Figure 1:**
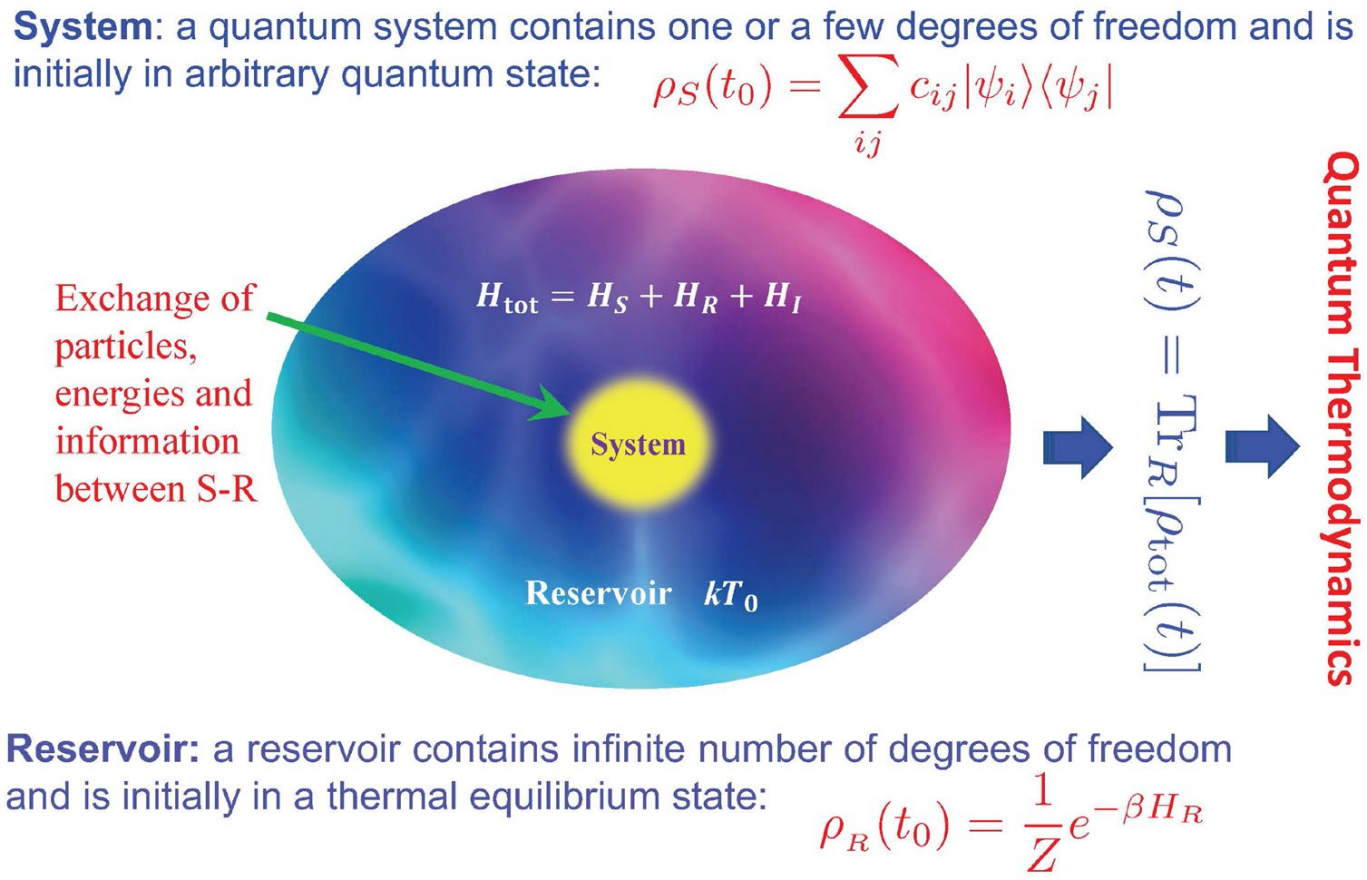
Schematic illustration of an open quantum system and thermalization. An open quantum system described by Hamiltonian $H_S$ is coupled to a reservoir with Hamiltonian $H_R$, and $H_{I}$ describes the interaction between the system and the reservoir. The reservoir is initially in a thermal equilibrium state with an initial temperature $T_0$ and *the system is initially in an arbitrary state * so that the initial total density matrix is $\rho _{\mathrm{tot}}(t_0)=\rho _S(t_0)\otimes \rho _{_R}(t_0)$. Then both the system and the reservoir evolve into non-equilibrium quantum states through the interaction Hamiltonian $H_{I}$, from which one can compute the reduced density matrix of the system and describe quantum thermodynamics under the principle of quantum mechanics.

As we have pointed out in the introduction, the big challenge in thermodynamics is the mechanism of thermalization. The solution to this problem relies on the deep understanding of the non-equilibrium time evolution of open quantum systems. A standard open quantum system coupled to reservoirs is described by the Hamiltonian,

6 $$\begin{aligned} H_{\mathrm{tot}} = H_S + H_R + H_I, \end{aligned}$$

which consists of the Hamiltonians of the system and the reservoirs, denoted respectively as $H_S$ and $H_R$, and the interaction between them, given by $H_I$, as shown schematically in Fig. 1. The dynamics of open quantum systems can be fully determined by the reduced quantum density matrix $\rho _S(t)$, which is defined by tracing over all the reservoir degrees of freedom from the total density matrix $\rho _{\mathrm{tot}}(t)$ of the system plus the reservoirs: $\rho _S(t)={{\,\mathrm{Tr}\,}}_R[\rho _{\mathrm{tot}}(t)]$. The total density matrix $\rho _{\mathrm{tot}}(t)$ obeys the von Neumann equation in the von Neumann formulation of quantum mechanics^39^ (sometime it is also called the quantum Liouville equation in the literature):

7 $$\begin{aligned} i\hbar {\dot{\rho }}_{\mathrm{tot}}(t)=[H_{\mathrm{tot}}, \rho _{\mathrm{tot}}(t)], \end{aligned}$$

from which we have the formal solution of the reduced density matrix:

8 $$\begin{aligned} \rho _S(t)= {{\,\mathrm{Tr}\,}}_R[U(t,t_0)\rho _{\mathrm{tot}}(t_0)U^\dag (t,t_0)]. \end{aligned}$$

Here $U(t,t_0)=\mathbf {T}\exp \{-\frac{i}{\hbar }\int _{t_0}^tdt'H_{\mathrm{tot}}(t')\}$ is the standard time-evolution operator in quantum mechanics which describes the full quantum dynamical evolution of both the system and reservoirs, where $\mathbf {T}$ is the time-ordering operator, and $\rho _{tot}(t_0)$ is the initial state of the total system (the system plus reservoirs). Solving Eq. (8) exactly and taking the asymptotic limit in time, one can find the answer to thermalization, as has been done using the Feynman-Vernon influence functional^40^ with initially decoupled system-reservoir state $\rho _{\mathrm{tot}}(t_0)=\rho _S(t_0) \times \rho _R(t_0)$ in the previous works for quantum Brownian motion^29,34,35^ and also in the recent works for a large class of open quantum systems^36–38^, which we have summarized in Introduction.

Notice that von Neumann reformulated and extended quantum mechanics in terms of the density matrix for quantum systems whose states can be either pure states or mixed states, motivated to develop both non-equilibrium quantum statistical mechanics and the theory of quantum measurements^39^. The two fundamental non-equilibrium quantum theories in the literature, the Schwinger-Keldysh’s nonequilibrium Green function approach^41–43^ and the Feynman-Vernon influence functional approach^40^ are established from the von Neumann formulation of quantum mechanics. The von Neumann formulation of quantum mechanics has now become the basis for open systems (systems coupled to reservoirs) in which the reservoir states are not pure states so that the Schrödinger formulation of quantum mechanics is inadequate to begin with.

With the previous achievements on thermalization^29,34–38^, we can develop quantum thermodynamics as follows. Within the von Neumann formalism of quantum mechanics, all physical observables of the system, such as energy, particle number, magnetic moment, etc. can be computed straightforwardly from the reduced density matrix $\rho _S(t)$ at any time *t* far from equilibrium:

9a $$\begin{aligned}&E(t) = {{\,\mathrm{Tr}\,}}_S [ \rho _S(t) H_S ], ~ N(t)={{\,\mathrm{Tr}\,}}_S[\rho _S(t) N_S], ~ \mathbf{M}(t) = {{\,\mathrm{Tr}\,}}_S[\rho _S(t)\mathbf{M}_S]~, \cdots . \end{aligned}$$

Also, the entropy which characterizes the information of the state $\rho _S(t)$ in the von Neumann formulation of quantum mechanics is given by

9b $$\begin{aligned} S(t) = - k {{\,\mathrm{Tr}\,}}_{S} \left[ \rho _S(t) \ln \rho _S(t) \right] . \end{aligned}$$

The von Neumann entropy is indeed the extension of Gibbs entropy to quantum mechanics, as von Neumann originally introduced to develop quantum statistical mechanics^39^. In the thermodynamical limit, the von Neumann entropy is identical to the Shannon entropy in the energy basis, and can correctly reproduce the thermodynamic entropy when the system reaches the equilibrium with the reservoirs.

Equation (9) establishes the fundamental relation among all the physical observables of the system through the quantum density matrix $\rho _S(t)$. This provides a general framework for the study of non-equilibrium quantum thermodynamics from non-equilibrium quantum statistical mechanics, in analogy to the equilibrium statistical mechanics of Eq. (5) which provides the microscopic basis for the equilibrium thermodynamics of Eqs. (1)–(3) summarized in Introduction. Now we can generalize the concepts of temperature, chemical potential, and magnetic moment, etc. into the regime far from equilibrium. We introduce the dynamical temperature ${{\mathcal {T}}}(t)$, dynamical chemical potential $\mathcal{\mu }(t)$, and external magnetic field $\mathbf{B}(t)$, etc. as the first derivatives of the dynamical energy of the system with respect to the dynamical entropy, particle number, and magnetic moment, etc. calculated from Eq. (9):

10 $$\begin{aligned} {{\mathcal {T}}}(t) \equiv \frac{\partial E(t)}{\partial S(t)}~, ~~ \mathcal{\mu }(t)\equiv \frac{\partial E(t)}{\partial N(t)}~,~~ \mathbf{B}(t)\equiv \frac{\partial E(t)}{\partial \mathbf{M}(t)},~ \cdots \end{aligned}$$

These dynamical thermal parameters evolve through various quantum states much before they approach to the steady state or thermal equilibrium with reservoirs. Conditionally, the dynamical thermal parameters defined by Eq. (10) can describe quantum thermodynamics if they are coincident to these equilibrium thermal parameters in the steady-state limit when the system dynamically approaches the thermal equilibrium with the reservoirs, as we will show in the next two sections. In fact, in the practical applications given in the next two sections, we will demonstrate explicitly that the dynamical temperature defined in Eq. (10) will always approach a non-negative thermodynamic temperature in the equilibrium limit for the weak system-reservoir couplings, as one expected.

Furthermore, in analogy to the axiomatic description of thermodynamics^26^, using Legendre transformation of *E*(*t*) with respect to *S*(*t*) that replaces the dynamical quantum entropy by the dynamical temperature as an independent variable, it natural gives the dynamical Helmholtz free energy far from equilibrium,

11 $$\begin{aligned} F(t) = E(t) - {{\mathcal {T}}}(t) S(t), \end{aligned}$$

which is a dynamical thermal potential resulting from the time evolution of the system. Equations (8–11) completes our quantum thermodynamics formalism far from equilibrium, in which all the physical observables are directly computable from the reduced density matrix which is determined fully from the principle of quantum mechanics. This novel theory of quantum thermodynamics is built on the non-equilibrium dynamics of open quantum systems.

In the literature, many efforts have been devoted to the problem of thermalization starting from an isolated quantum system under unitary evolution^1–5,20^. In conventional thermo-statistics, there are three equivalent statistical ensemble descriptions: the micro-canonical, canonical and grand-canonical ensembles. Both the canonical ensemble (which has the energy exchange with the reservoirs) and grand-canonical ensemble (has the energy and particle exchanges with the reservoirs) deal with open systems, as we have discussed in Introduction. Therefore, the steady-state limit of Eqs. (8–11) must reproduce the equilibrium statistical mechanics of canonical and grant-canonical ensemble statistics when the system dynamically approaches equilibrium with reservoirs, which guarantees the consistency of our formulation with the standard equilibrium thermodynamics.

On the other hand, the micro-canonical ensemble is defined for isolated systems with the hypothesis of equal probability for all the available microstates in which the energy, the volume and the particle number are fixed. The micro-canonical ensemble is usually considered as a pure mathematical model but it is fully equivalent to other two statistical ensemble description in practical statistical analysis. Physically, this is because for a macroscopic system, the energy level spacings of the system almost approach to zero due to the huge number of particles ($\sim 10^{23}$). On the other hand, the very weak but inevitable random gravitational and electromagnetic fields existing everywhere in nature. These very weak gravitational and electromagnetic interactions between all the particles, which may be negligible in dealing with individual particle dynamics, make enormously rapid random transition among various nearly degenerated quantum states in macroscopic systems. This results in the realization of equal probability hypothesis in reality, as analyzed in details in Ref.^44^. In other words, no physical system is, or even can be, truly isolated^44^.

Meantime, for an ideal isolated quantum system, if its quantum state is initially in a pure state, the unitary evolution makes the system evolving always in pure states so that no thermalization can happen. In order to make thermalization possible in reality, it is necessary to prepare the isolated system initially in a mixed state or drive the system out of equilibrium by quench. This is just a special case of Eq. (7) in which no trace should be taken (because of the isolated system) so that the time-evolution of the quantum density matrix remains unitary but the quantum state must be expressed in terms of the density matrix and evolution in a controllable way. Thus, our formulation of quantum thermodynamics covers the studies of thermalization for quenched isolated systems as well.

Furthermore, in equilibrium thermodynamics, only for isothermal process one can define the thermodynamic work as the change of the Helmholtz free energy^26^. The thermodynamic heat can be obtained thereby as a consequence of the energy conservation including the energy transfer between the system and reservoirs. However, for quantum systems under dynamical evolution, no process is isothermal and therefore the extension of the concept of work far from equilibrium is not straightforward. In fact, in the framework of quantum mechanics, no concepts of work and heat have been introduced because work and heat are not quantum mechanical observables. We expect that by coupling the system with the environment, it is possible to explore the microscopical quantum origins of work and heat from the dynamics of open quantum systems, which are manifested by dissipation dynamics and fluctuation dynamics, respectively. We will explore these fundamental concepts in the further research within our exact master equation theory^21–25^.

It is also worth mentioning that currently there is also no consensus on the definition of the internal energy for systems strongly coupled with reservoirs^45–48^. Some inconsistency has been found in calculating the internal energy in terms of the local system Hamiltonian but usually such inconsistency is mainly due to the approximations used in dealing with the dynamics of open quantum systems^10^, namely, lack of the exact solution of Eq. (8). It has also been shown that by identifying the thermodynamic internal energy with the equilibrium expectation value of the local system Hamiltonian $H_S$ for a damped particle, the resulting specific heat approaches the classical result for high temperatures and goes to zero for vanishing temperature in accordance with the third law at equilibrium^45^.

Nevertheless, the key to solve quantum thermodynamics in this new formulation is the determination of the time-dependent reduced quantum density matrix of Eq. (8) from the non-equilibrium dynamical evolution of both the system and reservoirs under the total Hamiltonian of Eq. (6), and from which the non-equilibrium quantum thermodynamics of the system can be unambiguously investigated. To obtain unambiguous consequences, in the following, we shall apply the theory to a single-mode cavity system and a two-level atomic system interacting with a reservoir, which are described by the Fano-Anderson model^49,50^ and the multimode Jaynes-Cummings (JC) model^51,52^, respectively. The Fano-Anderson model gives the Fano resonance and Anderson localization observed as universal phenomena throughout nuclear, atomic, molecular, and optical physics, as well as in various condensed matter systems^53,54^, while the JC model is the basis of cavity QED, describing the light-matter interaction in quantum optics^55,56^. These are the two most general examples that can be solved unambiguously in open quantum systems.

In these applications, we shall show rigorously and exactly (i) how thermodynamics emerges from quantum dynamics of a single particle system coupled to a reservoir in the weak system-reservoir coupling regime, without introducing the hypothesis of equilibrium between the system and the reservoir from the beginning; (ii) how thermodynamics also emerges in the intermediate system-reservoir coupling regime where the Born-Markovian approximation is broken down; (iii) how the thermodynamics breaks down in the strong coupling regime, due to the long-time non-Markovian memory effect arisen from the occurrence of localized bound states; and (iv) how a dynamical quantum phase transition occurs associated with negative temperature when the reservoir is initially in vacuum state or its *initial* thermal energy $kT_0$ is less than the system initial energy $E(t_0)$. The dynamical criticality in this dynamical quantum phase transition provides an inflationary dynamics that may reveal a simple physical picture to the origin of universe inflation^57^. We also show how the third law of thermodynamics, as a true quantum effect, is obtained in this new formulation of quantum thermodynamics.

### Application to cavity systems with Fano-Anderson model

We first consider the system as a single-mode bosonic system, such as a single-mode cavity, with $H_S=\hbar \omega _s a^{\dagger } a$, coupled with a heat bath (it is also applicable to fermionic system in a similar way^22,23^). Here $\omega _s$ is the frequency of a single-mode boson. The bath is modeled as a collection of infinite bosonic modes with a continuous frequency spectrum, and the system-bath interaction is described by the Fano-Anderson model that has wide applications in nuclear, atomic, molecular, and optical physics, as well as in various condensed matter systems^53,54^. Thus, the total Hamiltonian of the system plus the bath is given by the Fano-Anderson Hamiltonian,

12 $$\begin{aligned} H_{\mathrm{tot}}= \hbar \omega _s a^\dag a + \sum _k \hbar \omega _k b^\dag _k b_k + \sum _k \hbar (V_k a^\dag b_k + V^*_k b^\dag _k a) . \end{aligned}$$

The reduced quantum density matrix of the system obey the following exact master equation^21,58,59^:

13 $$\begin{aligned} \frac{d}{d t} \rho _S(t) =&-i \omega _s^{\prime }(t,t_0)\big [a^\dag a , \rho (t)\big ] + \gamma (t,t_0) \big [2a \rho _S(t) a^{\dag } -\! a^{\dag } a \rho _S(t) \! -\! \rho _S(t) a^{\dag }a\big ] ~ \nonumber \\&+{\widetilde{\gamma }}(t,t_0) \big [a^{\dag }\rho _S(t) a + a\rho _S(t) a^{\dag } - a^{\dag }a\rho _S(t) \! -\! \rho _S(t) a a^{\dag }\big ], \end{aligned}$$

in which the renormalized system frequency, the dissipation (relaxation) and fluctuation (noise) coefficients are determined simply by $\omega _s^{\prime }(t,t_0) = -{\mathrm{Im}}[{\dot{u}}(t,t_0)/u(t,t_0)]$, $\gamma (t,t_0) = -{\mathrm{Re}}[{\dot{u}}(t,t_0)/u(t,t_0)]$ and ${\widetilde{\gamma }}(t,t_0)={\dot{v}}(t,t)-2v(t,t){\mathrm{Re}}[{\dot{u}}(t,t_0)/u(t,t_0)]$, where the non-equilibrium Green’s’ functions satisfy the following equations

14a $$\begin{aligned}&\frac{d}{dt}u(t,t_0) + i \omega _s u(t,t_0) + \int _{t_0}^t d\tau g(t,\tau ) u(\tau ,t_0) = 0. \end{aligned}$$

14b $$\begin{aligned}&v(t,t)=\int _{t_0}^t d\tau _1 \int _{t_0}^t d\tau _2 ~ u(t,\tau _1) {{\widetilde{g}}}(\tau _1,\tau _2)u^{*}(t,\tau _2), \end{aligned}$$

The integral kernels $g(t,\tau ) = \int _0^{\infty } d\omega J(\omega ) e^{-i\omega (t-\tau )}$ and ${{\widetilde{g}}}(\tau _1,\tau _2)\!=\!\int _0^{\infty } d\omega J(\omega ) {{\overline{n}}}(\omega ,T_0) e^{-i\omega (\tau _1-\tau _2)}$ are determined by the spectral density $J(\omega ) \equiv \sum _k |V_k|^2 \delta (\omega -\omega _k)$ of the reservoir, and $\overline{n}(\omega ,T_0)=\frac{1}{e^{\hbar \omega / k T_0}-1}$ is the initial particle number distribution of the reservoir. The exact analytic solution of the non-equilibrium Green’s function $u(t,t_0)$ has been given^21^,

15 $$\begin{aligned} u \left( t , t _{0} \right) =&\frac{1}{1 - \Sigma ^{\prime } \left( \omega _{b} \right) } e ^{- i \omega _{b} \left( t - t _{0} \right) } + \int _{0} ^{\infty } \!\!\!d \omega \frac{J \left( \omega \right) e ^{- i \omega \left( t - t _{0} \right) }}{\left[ \omega - \omega _{s} - \Delta \left( \omega \right) \right] ^{2} + \pi ^{2} J ^{2} \left( \omega \right) } , \end{aligned}$$

where $\omega _b$ is the localized photon mode frequency determined by the pole condition: $\omega _{b} - \omega _{s} - \Sigma \left( \omega _{b} \right) = 0$ with the self-energy $\Sigma \left( z \right) = \int d \omega ^{\prime } \frac{J \left( \omega ^{\prime } \right) }{z - \omega ^{\prime }}$, from which we have $\Sigma \left( \omega \pm i 0 ^{+} \right) = \Delta \left( \omega \right) \mp i \pi J \left( \omega \right)$, and the principle value $\Delta \left( \omega \right) = {{\mathcal {P}}}\int d \omega ^{\prime } \frac{J \left( \omega ^{\prime } \right) }{z - \omega ^{\prime }}$ is the frequency shift. The first term in the above solution is the contribution of dissipationless localized bound state. The second term is the continuous part of the spectra (corresponding to the level broadening induced by the reservoir) that characterizes the relaxation of the system. The Green’s function *v*(*t*, *t*) which characterizes the non-equilibrium thermal fluctuations gives the non-equilibrium fluctuation-dissipation theorem^21^. Without loss of generality, we consider an Ohmic spectral density $J(\omega ) = \eta \omega \exp \left( -\omega /\omega _c \right)$, where $\eta$ is the coupling strength between the system and the thermal reservoir, and $\omega _c$ is the frequency cutoff of the reservoir spectra^60^. In this case, the system has a localized bound state when the coupling strength $\eta$ exceeds the critical value $\eta _c=\omega _s/\omega _c$^21^. Then, all quantum thermodynamical parameters can be calculated systematically and exactly.

Explicitly, let the system be prepared in a Fock state $|n_0\rangle$, the corresponding initial density matrix $\rho _S(t_0)= |n_0 \rangle \langle n_0|$, with initial system energy $E_0 = {n_0} \hbar \omega _s$ and ${n_0}$ is an integer. The reservoir is initially in thermal equilibrium state with an initial temperature $T_0$, as a condition for searching the emergence of thermal equilibrium of the system. After the initial time $t_0$, both the system and the reservoir evolve into non-equilibrium states due to the interaction between the system and the reservoir. The system state at arbitrary later time *t* can then be obtained rigorously by solving the exact master equation of Eq. (13). The result is^37^:

16a $$\begin{aligned}&\rho _S(t) = \sum _{n=0}^{\infty } p^{n_0}_n(t) |n \rangle \langle n|, \end{aligned}$$

16b $$\begin{aligned}{\,}&p_{n} ^{n _{0}} (t) = \frac{[ v (t,t)] ^{n}}{[ 1 + v (t,t) ] ^{n + 1}} \left[ 1 - A \left( t,t_0 \right) \right] ^{n _{0}} \nonumber \\& \times \!\!\!\!\!\!\sum _{k=0}^{\mathrm{min\{n_0,n\}}}\!\!\!\Bigg (\!\!\begin{array}{c} n_0 \\ k \end{array}\!\!\Bigg ) \Bigg (\!\!\begin{array}{c} n \\ k \end{array}\!\!\Bigg )\Bigg [\frac{1}{v(t,t)}\frac{A(t,t_0)}{1-A(t,t_0)} \Bigg ]^k , \end{aligned}$$

where $A \left( t,t_0 \right) = \frac{\left| u \left( t , t _{0} \right) \right| ^{2}}{1 + v \left( t , t \right) }$, and the explicit solutions of $u(t,t_0)$ and *v*(*t*, *t*) are given by Eqs. (15) and (14b). It shows that the system in an initial photon Fock state will evolve into a mixed state of different Fock states due to the coupling to the reservoir, and the function $p^{n_0}_n(t)$ is the probability of finding the system in the Fock state $|n\rangle$ at time *t*. The probability $p^{n_0}_n(t)$ is fully determined by the initial state of the system and the non-equilibrium Green’s functions $u(t,t_0)$ and *v*(*t*, *t*). The different initial state will lead to the different probability distribution in this fully quantum mechanical non-equilibrium evolution. On the other hand, the dependence of $u(t,t_0)$ and *v*(*t*, *t*) fully characterizes the dissipation and fluctuation dynamics between the system and the reservoir during the non-equilibrium evolution. From these results, we can calculate straightforwardly all the quantum thermodynamic parameters, namely the dynamical energy, entropy, temperature and free energy, etc. Explicitly, the internal energy of the system at time *t*, i.e. Eq. (9a), is given by $E(t) = \sum _n p^{n_0}_n(t) E_n$ with $E_n=n\hbar \omega _s$. The von Neumann entropy of the reduced density operator $\rho (t)$, i.e., Eq. (9b), is simply reduced to $S(t) = - k \sum _{n=0}^{\infty } p^{n_0}_n(t) \log p^{n_0}_n(t)$. These results provide explicitly the fundamental relation between the quantum energy and the quantum entropy of the system, from which one can determine the dynamical temperature ${{\mathcal {T}}}(t)$ defined by Eq. (10), and all other related quantum thermodynamical parameters. The results are given as follows.

#### Emergence of thermodynamics and thermo-statistics from quantum dynamics in the weak system-reservoir coupling limit

As it is well-known, classical statistical mechanism is valid for relatively high temperatures. This is because at relatively high temperatures, the Fermi-Dirac distribution and the Bose-Einstein distribution are reduced to the classical Boltzmann distribution. At low temperatures, the Fermi-Dirac distribution and the Bose-Einstein distribution can be significantly different from the classical Boltzmann distribution, and then classical statistical mechanics fails. The specific heat of metal at low temperatures provides a prototype example for such an invalidity of classical statistical mechanics^26^. However, in general, it is not clear what a temperature value can be regarded as high temperature. Here we define quantitatively this classical (i.e. the high temperature) regime: $kT_0 > E_0$, namely the initial thermo-energy of the reservoir should be larger than the initial energy of the system. In this classical regime, it is indeed straightforward to see how the system is dynamically thermalized in the weak system-reservoir coupling limit $(\eta \ll \eta _c)$. Initially the system is at zero temperature (because it is in the pure state $|n_0\rangle$) with zero entropy, and the reservoir is in thermal equilibrium at a relatively high temperature $T_0$ with $kT_0 > E_0=n_0\hbar \omega _s$. Then the system and the reservoir undergo a non-equilibrium process due to their contact with each other. The non-equilibrium quantum dynamics is fully determined by the non-equilibrium Green functions $u(t,t_0)$ and *v*(*t*, *t*) of Eq. (14). One can indeed show explicitly that in the long time steady-state limit $t\rightarrow t_s$, we have $u(t_s, t_0) \rightarrow 0$, and the average photon number in the cavity obeys the Bose-Einstein statistical distribution,

17 $$\begin{aligned} \langle a^\dag (t_s)a(t_s)\rangle = v(t_s, t_s) \rightarrow \overline{n}(\omega _s,T_0) =\frac{1}{e^{\hbar \omega _s/kT_0}-1} . \end{aligned}$$

The probability $p^{n_0}_n(t \rightarrow t_s)$ of occupying the *n*-th energy state is then solely determined by the Bose-Einstein statistical distribution, $\overline{n}(\omega _s,T_0)$, and the state of the system ultimately approaches unambiguously to the thermal equilibrium with the reservoir^36,37^

18 $$\begin{aligned} \rho _S(t\rightarrow t_s) = \sum _{n=0}^{\infty } \frac{[\overline{n}(\omega _s,T_0)]^n}{[1+\overline{n}(\omega _s,T_0)]^{n+1}} |n\rangle \langle n | =\frac{1}{Z}e^{-\frac{H_S}{k T_0}}, \end{aligned}$$

where $Z={{\,\mathrm{Tr}\,}}_S[\exp (-H_S/{kT_0})]$, and $T_0$ is the initial temperature of the reservoir. Without taking any approximation in the above calculations, we provide for the first time a rigorous proof of the dynamical thermalization within the quantum mechanical framework.

**Figure 2:**
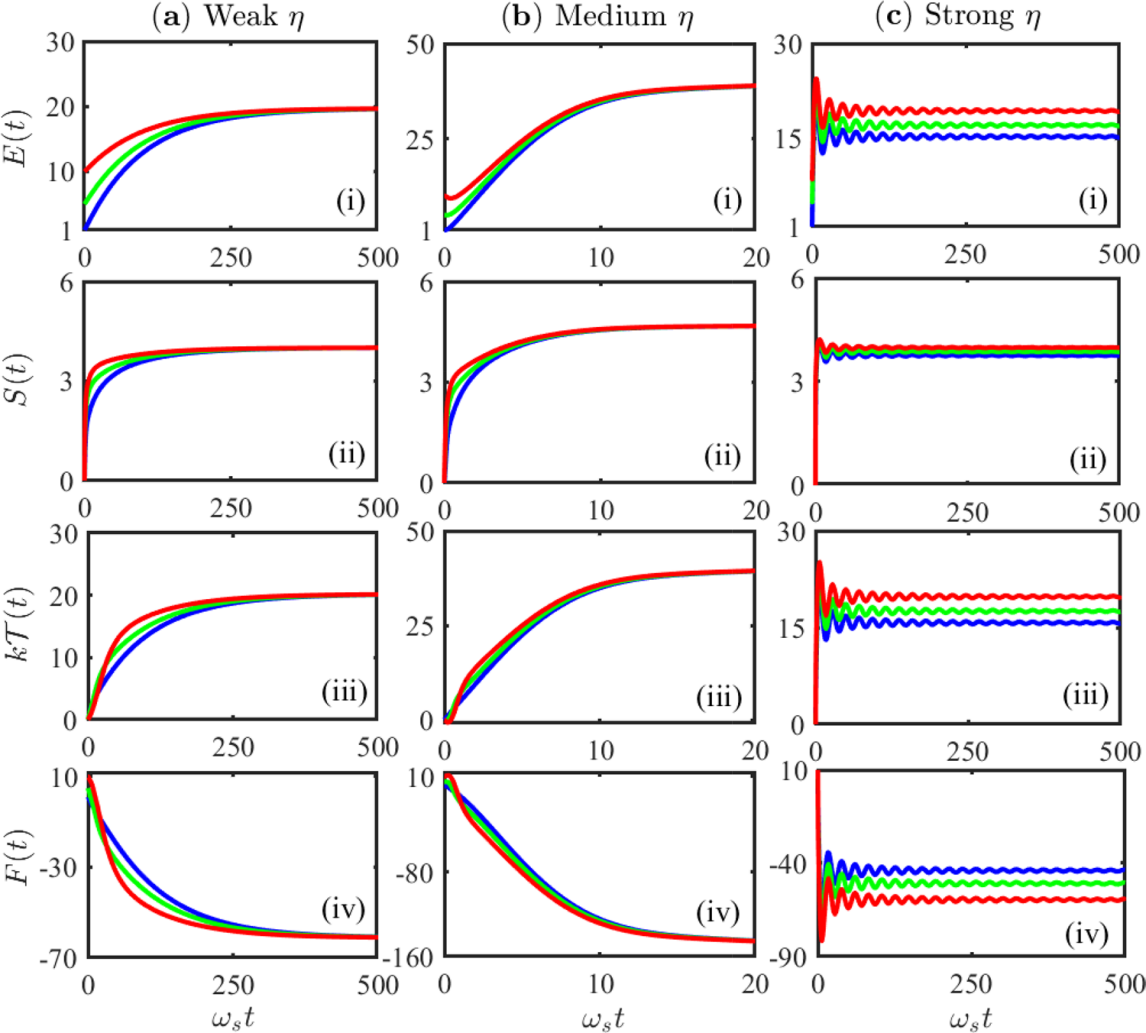
Non-equilibrium quantum thermodynamics in different system-reservoir coupling regimes. The dynamics of non-equilibrium thermal parameters: the energy, the entropy, the temperature and the free energy (in the unit of $\hbar \omega _s$, except for the entropy which is dimensionless when the temperature takes the same unit as the energy) as a function of the time that calculated from Eqs. (9)–(11) with different initial Fock states: $|n_0\rangle$ of $n_0=1$ (blue), $n_0=5$ (green) and $n_0=10$ (red). **(a)** The weak coupling regime $\eta =0.01 \eta _c \ll \eta _c$ (the first column): **(b)** The intermediate coupling range $0.01\eta _c<\eta =0.5 \eta _c<\eta _c$ (the second column): **(c)** The strong coupling regime $\eta =1.5 \eta _c > \eta _c$ (the third column). The initial temperature of the thermal reservoir is taken at $kT_0=20 \hbar \omega _s$, the cut-off frequency $\omega _c=5\omega _s$.

One can then calculate the dynamical quantum internal energy and the quantum entropy. In Fig. 2a, we show the dynamics of internal energy *E*(*t*), entropy *S*(*t*), temperature ${{\mathcal {T}}}(t)$ and free energy *F*(*t*) as a function of time in the weak system-reservoir coupling regime for different initial states of the system. As we see, both the energy and entropy of the system increases with time due to its contact with the thermal reservoir through a non-equilibrium process in the classical regime ($kT_0 > E_0$), see Fig. 2a(i) and 2**a**(ii). The dynamical temperature ${{\mathcal {T}}}(t)$ of the system increases gradually (see Fig. 2a(iii)) and finally approaches to the temperature that is identical to the initial temperature of the reservoir, ${{\mathcal {T}}}(t_s)=T_0$, namely the system reaches the thermal equilibrium with the reservoir.

**Figure 3:**
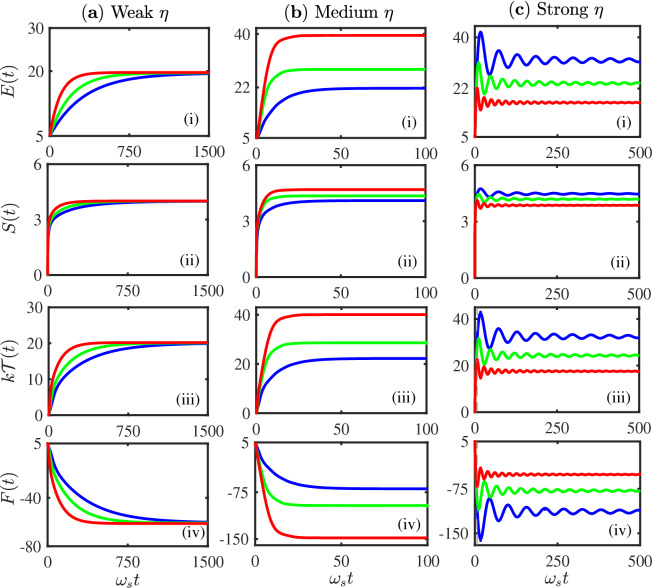
Non-equilibrium quantum thermodynamics in different system-reservoir coupling regimes. The dynamics of non-equilibrium thermal parameters: the energy, the entropy, the temperature and the free energy (in the unit of $\hbar \omega _s$, except for the entropy which is dimensionless when the temperature takes the same unit as the energy) as a function of the time that calculated from Eqs. (9)–(11) for different system-reservoir coupling strengths with the same initial Fock state $|n_0\rangle = |5\rangle$. (**a**) The weak coupling range $\eta \le 0.01\eta _c$ (the first column): three different curves represent the three different coupling strengths are $\eta =0.003\eta _c$ (blue), $\eta =0.005\eta _c$ (green), $\eta =0.01\eta _c$ (red). (**b**) The intermediate coupling range $0.01\eta _c<\eta <\eta _c$ (the second column): three different curves represent the different coupling strengths $\eta =0.1\eta _c$ (blue), $\eta =0.3\eta _c$ (green), $\eta =0.5\eta _c$ (red). (**c**) The strong coupling regime $\eta > \eta _c$ (the third column): three different curves represent the different coupling strengths $\eta =1.2\eta _c$ (blue), $\eta =1.3\eta _c$ (green), and $\eta =1.5\eta _c$ (red). The initial temperature of the thermal reservoir is taken at $kT_0=20 \hbar \omega _s$, the cut-off frequency $\omega _c=5\omega _s$.

The results in Fig. 2a show precisely how thermalization is dynamically realized in the weak system-reservoir coupling limit for a single cavity system, *independent of its initial states*. As it is expected, the entropy always increases in non-equilibrium processes, see Fig. 2a(ii), demonstrating the second law of thermodynamics. The second law places constraints on state transformations^61^, according to which the system evolving non-equilibriumly from the initial state $\rho _S(t_0)$ makes the free energy *F*(*t*) goes down, as shown in Fig. 2a(iv). Meantime, the increasing internal energy with the decreasing free energy always makes the heat transfer *into* the system (non-equilibrium extension of the first law of thermodynamics), resulting in the increase of the dynamical temperature ${{\mathcal {T}}}(t)$ of the system, as shown in Fig. 2a(iii). In the steady-state limit, the free energy approaches to a minimum value with maximum entropy in the equilibrium state, which justifies the fundamental equation of the equilibrium thermodynamics^26^. Therefore, this solution in the weak system-reservoir coupling regime provides the foundation of the equilibrium thermodynamics, namely how thermalization is dynamically realized within the framework of a fully dynamical evolution of quantum mechanics. Indeed, *producing the equilibrium thermodynamics in the classical regime from the exact quantum dynamics with arbitrary initial states of the system should also be considered as a criterion for an unambiguous formulation of quantum thermodynamics.*

#### Emergence of thermodynamics in the intermediate system-reservoir coupling regime that goes beyond the Born-Markov limit

It may be worth pointing out that the above thermalization in the weak-coupling limit can also be obtained approximately from the Born-Markov master equation. However, the Born approximation requires the reservoir to remain in the unchanged equilibrium state all the time which is indeed an inconsistent assumption. Specifically, if one consider an interesting situation in which the system interacts very weakly with two same thermal reservoirs with different initial temperatures, say $T_1$ and $T_2$, respectively. Note that here the two reservoirs plus the systems form a closed system, namely no external thermal sources in contact with to maintain the two reservoirs with their initial temperatures $T_1$ and $T_2$, respectively. Then after a sufficient long time, the system and the two reservoirs must eventually reach the same equilibrium state with a same new temperature *T*. In other words, both reservoirs cannot be kept unchanged in time so that the basic assumption of the Born-Markov approximation is no longer valid, even in the very weak-coupling regime. However, our theory describes exactly the non-equilibrium dynamical evolution of the whole system (the system and also the two reservoirs). The exact solution of our formulation naturally shows how the system and the two reservoirs evolve into a new equilibrium state at the same time, with the new equilibrium temperature $T=(T_1+T_2)/2$, which also demonstrates the zero-th law of thermodynamics.

Furthermore, we show next that in the intermediate system-reservoir coupling regime, thermodynamics can still be reached where the Born-Markov approximation is completely broken down. In fact, we find that the thermalization does not crucially rely on the assumption of the very weak interaction between the system and the reservoir. In the literature, an overall thermalization condition on the system-reservoir coupling was proposed^62^: the strength of the coupling $\eta$ induces the effect that has to be negligible in comparison to initial thermal energy $kT_0$. Recently, many efforts have been made to formulate quantum thermodynamics for a strongly coupled system^36,37,63–69^. However, quantum thermodynamics has not been fully solved from a quantum dynamics description in the strong system-reservoir coupling regime.

From our solution of the exact master equation, we can provide an exact quantum dynamics description in the strong system-reservoir coupling regime, from which we can give a quantitative condition on thermalization. The result is that thermalization occurs when $\eta < \eta _c$, where $\eta _c$ is the critical value of the system-reservoir coupling for the occurrence of localized bound states^21,36,37,58,59^. In particular, our numerical calculation shows that the Born-Markov thermalization condition is valid only for the very weak coupling $\eta \lesssim 0.01 \eta _c$. In Fig. 3a, we plot the non-equilibrium thermodynamical parameters in the very weak-coupling regime for several different system-reservoir coupling strengths with the same initial system state. It shows that the system reaches the same equilibrium state in the steady-state limit. In other words, in the very weak-coupling regime, the change of the coupling strength $\eta$ induces the effect that is negligible in comparison to initial thermal energy $kT_0$, as proposed in Ref.^62^. However, we find that this is only a sufficient but not a necessary condition for thermalization.

We show below that quantum thermodynamics in the strong system-reservoir coupling regime is indeed quite distinct. As it is shown in Fig. 2b, when the system-reservoir coupling strengths are not so weak (the intermediate range: $0.01 \eta _c< \eta < \eta _c$), the system quickly approaches to the same equilibrium state in the steady-state limit for different initial states but the same coupling strength. In other words, the system reaches the equilibrium state which is independent of the initial state of the system as well in the intermediate coupling strength regime. However, for different intermediate coupling strengths, the system approaches to different equilibrium states in the steady-state limit, as shown in Fig. 3b. We called these different equilibrium states as thermal-like states in Ref.^36^) because these thermal states are characterized by the different steady-state temperatures that are all different from the initial reservoir temperature $T_0$ (see the blue, green and red curves in Fig. 3b(iii), which correspond to different system-reservoir coupling strengths). In this intermediate coupling strength region, different equilibrium states are reached by different steady-state minima of the free energy *F*(*t*), see Fig. 3b(iv).

These results already show the deviations of quantum thermodynamics away from the equilibrium thermodynamics prediction, namely after a long enough time, the system must equilibrate with the reservoir that is characterized by the initial reservoir temperature $T_0$. In fact, due to the relatively stronger couplings between the system and the reservoir, both the system and the reservoir undergo a non-equilibrium process, and then approach to a new common equilibrium state characterized by the new equilibrium temperature ${{\mathcal {T}}}(t \rightarrow t_s)$ that is different from the initial reservoir temperature $T_0$, as our theory predicted. In Figs. 2b and 3b, it shows that $t_s \simeq 25/\omega _s$, and ${{\mathcal {T}}}(t_s)$ changes as $\eta$ varies. But the principle of thermodynamics, namely the existence of equilibrium state and the associated thermodynamic laws in the steady state, is still valid, and the results are independent of the initial states of the system. In other words, thermalization can still be reached in the intermediate coupling regime, although the final equilibrium temperature of both the system and the reservoir is different from the initial temperature of the reservoir, namely the Born-Markovian approximation has been completely broken down here. This solution also shows explicitly that the thermalization can be realized far beyond the condition proposed in^62^.

#### Breakdown of thermodynamics and thermo-statistics due to the occurrence of localized bound states

We consider next the strong system-reservoir coupling regime (going beyond the critical coupling value $\eta > \eta _c$, where localized bound states occur in the system). In such strong coupling regime, the system indeed does not approach to thermal equilibrium^36^, because all different initial states result in different steady states, as shown explicitly in Fig. 2c. Furthermore, the average energy *E*(*t*) of the system initially increases, and then it starts decreasing with time, indicating a backflow of energy from the system into the reservoir, and finally it goes a stationary oscillation in time, see Figs. 2c(i) and 3c(i). Therefore, these steady states cannot be equilibrium states because of their permanent time dependence. A similar dynamical behavior of the system entropy *S*(*t*) is seen in Figs. 2c(ii) and 3c(ii) in this strong coupling regime, namely, although the entropy increases in the beginning, it is decreased and oscillates in the later time. Thus, the occurrence of localized bound states in the strong-coupling regime makes the steady state of the system deviated significantly away from the equilibrium state, and thermalization cannot be reached. Also, one does not see from Figs. 2c(iii) and 3**c**(iii) a monotonous increase of dynamical temperature ${{\mathcal {T}}}(t)$ that can eventually approach to a constant value. In other words, in the strong coupling regime with the occurrence of localized bound states, the system cannot reach to a thermal equilibrium state, it indeed approaches to a more complex non-equilibrium (oscillatory) steady state^36^. Under this situation, the non-equilibrium free energy *F*(*t*) also shows nontrivial oscillations with local minima, instead of having a global minimum, as shown in Figs. 2c(iv) and 3c(iv).

The physical origin for the breakdown of thermodynamics in the strong system-reservoir coupling regime with the occurrence of localized bound states comes from the important effect of long-time non-Markovian memory dynamics, as we have discovered earlier^21,36,58,59,70,71^. Explicitly, the general solution of the non-equilibrium Green function $u(t,t_0)$ in the long time limit is reduced to a partially dissipationless state

19 $$\begin{aligned} u(t \rightarrow \infty ,t_0)= \frac{1}{1 - \Sigma ^{\prime } \left( \omega _{b} \right) } e ^{- i \omega _{b} \left( t - t _{0} \right) }, \end{aligned}$$

which is very different from the solution in the long time limit for the case of $\eta < \eta _c$ where $u(t \rightarrow \infty ,t_0)=0$ (complete dissipation). Thus, the reduced density matrix of the system, given by Eq. (16) cannot be reduced to a thermal state, it will maintain the same form of Eq. (16) in the long time limit so that its steady-state limit is always dependent on the initial state. In this situation, the system can memorize forever some of its initial state information, i.e. initial state information cannot be completely wiped out through the thermal fluctuations from the reservoir. Therefore, the basic hypothesis of statistical mechanics, *namely after a sufficient long enough time, the system will evolve into an equilibrium state with the reservoir that its distribution is independent of its initial state*, breaks down completely and the system is unable to be thermalized. The oscillation behavior in Figs. 2c and 3c is a manifestation of non-Markovian memory effect originating from the localized bound state of the system, a general non-Markovian dynamic feature in open quantum systems^21^. The localized bound states of the system produces dissipationless dynamics, see Eq. (19), that do not allow the system to be thermalized with its environment, which was indeed noticed earlier by Anderson^49,54^ and is justified recently by one of us^36^. The localized bound state can appear when the system-environment coupling strength exceeds some critical value $\eta _c$ that is determined by the structure of spectral density $J(\omega )$. For the Ohmic spectral density, $\eta _c=\omega _s/\omega _c$. This solution also reveals the fact that the transition from thermalization to localization can occur not only in many-body systems^72^, but dynamically also in single particle quantum systems coupled with reservoirs^73^. However, it must be pointed out that for some spectral densities, such as the Lorentz spectral density considered in the next section, there will exist no localized bound state no matter how strong the coupling strength is^21^. In these cases, the system can always reach to an equilibrium state with the reservoir even in the strong coupling regime, and the results show the same behavior as demonstrated in Sec. IIIB.

#### Dynamical quantum phase transition and quantum criticality with quantum-classical border

**Figure 4:**
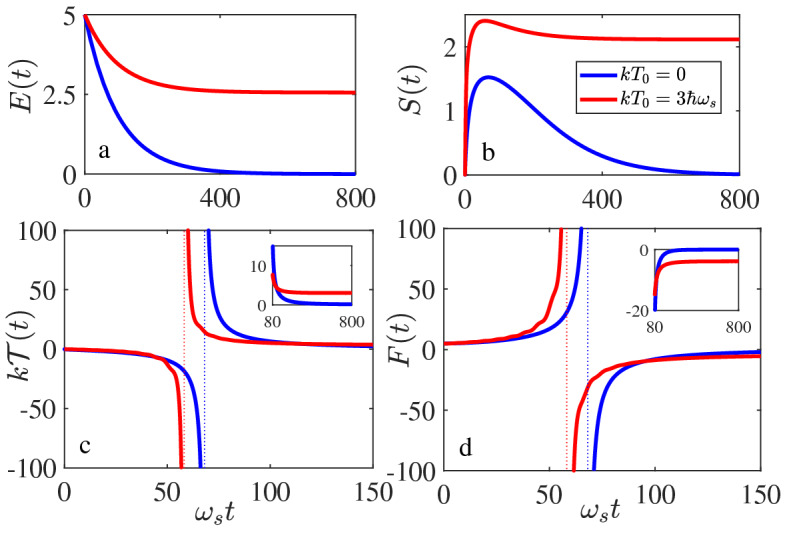
Inflationary dynamics and dynamical quantum phase transition. The dynamics of (**a**) energy *E*(*t*), (**b**) entropy *S*(*t*), (**c**) dynamical temperature ${{\mathcal {T}}}(t)$, and (d) free energy *F*(*t*) (in the unit of $\hbar \omega _s$, except for the entropy as specified in Figs. 2 and 3) are shown for the reservoir initially in vacuum state (blue curves) and for the reservoir with initial thermal energy $kT_0=3 \hbar \omega _s$ (red curves). The system is initially in the state $|n_0\rangle =|5\rangle$ with the initial energy $E_0=5 \hbar \omega _s$, and a weak system-reservoir coupling strength $\eta =0.01\eta _c$ is taken.

Now we discuss a very interesting situation occurring in the weak-coupling quantum limit in the quantum regime. *The quantum regime is defined as the situation in which the initial thermal energy of the reservoir is less than the system initial energy:*$kT_0 < E_0$. In this situation, the energy dissipation dominates the non-equilibrium process so that the system energy always decreases in time (see Fig. 4a), which is very different from the classical regime in Figs. 2a and 3a where the reservoir is in a relatively high temperature and the average energy flows between the system and the reservoir in an opposite direction.

On the other hand, as shown in Fig. 4b the system entropy (beginning with zero) will increase to a maximum value at an intermediate time, then the entropy turns to decrease and eventually approaches to zero (the blue curve in Fig. 4b for $kT_0=0$). This phenomenon can happen because the system is initially in a pure state (zero entropy), then the system takes dissipation (relaxation) and goes eventually into the vacuum state (back to a pure state) when all energy of the (small) system is dissipated into the (large) reservoir. Thus, at the beginning and in the end of the time-evolution process, the entropy of the system is zero. Therefore starting from the beginning, the entropy must go up (increase) in time and reach the maximum at some intermediate time (corresponding to the maximizing mixed state of the system), and henceforth go down (decrease) to zero again. On the other hand, because the system energy always decreases, the dynamical temperature which starts from zero, must decrease to negative infinity as the entropy approaches to the maximum. It then jumps from the negative infinite temperature to the positive infinite temperature at the maximum entropy point, after that the temperature goes down as the entropy decreases, and eventually reaches to zero temperature at the steady state, as shown by the blue curve in Fig. 4c.

This dramatic change of the dynamical temperature shows that in the quantum regime the time-evolution of the system could break the second law of thermodynamics for the system alone (because of the appearance of a negative temperature and the decrease of the entropy in the non-equilibrium regime), but the system plus the reservoir together still obey the second law. Also at the end, the entropy and the temperature both approach to zero, as a manifestation of the third law of thermodynamics in equilibrium state. Note that the third law of thermodynamics is hypothesized in equilibrium thermodynamics, it cannot be demonstrated within equilibrium thermodynamics, namely the zero temperature is not reachable as the entropy cannot approach to zero in the classical regime. In fact, zero temperature locates in the very deep quantum realm (corresponding to the situation of the system staying in the ground state). Therefore as a criteria, the theory of quantum thermodynamics must be able to capture the generalized third law of thermodynamics, namely the entropy becomes zero as the temperature approaches to zero in the deep quantum regime. Here we show explicitly how the third law of thermodynamics at equilibrium can be obtained dynamically from quantum mechanics.

On the other hand, the negative temperature in open quantum systems we find here only appears in the non-equilibrium region. We find that this negative dynamical temperature phenomenon occurs as long as the system is initially in a pure state with the condition that the system initial energy $E_0$ is large than the initial thermal energy $kT_0$ of the reservoir, namely in the quantum regime. More importantly, we find that the occurrence of the negative non-equilibrium temperature is accompanied with a nontrivial behavior of the entropy changing from increasing to decreasing, as shown in Fig. 4b, c. Here we would like to make a conjecture that this unusual dynamics may relate to the origin of the universe inflation^57^. The experimental observations show that the universe is still accelerating expansion and the average temperature of the universe today is approximately 2.73 K^74^. Thus the universe can be considered as an open system^75^. As an open system, we may propose that in the very beginning (before the big bang), the universe is a single quantum system staying in a pure quantum state^76^ and nothing else. In other words, one can assume that there is a vacuum reservoir for the universe in the very beginning. Then the universe might evolve through a non-equilibrium process similar to that given in Fig. 4. The sudden jump from the negative infinite temperature to the positive infinite temperature at the transition point in Fig. 4c (corresponding to the entropy varies from increase to decrease in time) allows the system to suddenly generate an inflationary amount of heat energy, as a result of the maximizing information (entropy) of the system. At the same time, the free energy of the system is always increased during the non-equilibrium process, but only at the transition point it is suddenly dropped. This sudden change delivers an inflationary energy to the reservoir, see Fig. 4d. This may provide a simple physical process for the origin of big bang and the universe inflation^57,76^ which is a long-standing problem that has not been solved so far in quantum cosmology. The continuous decrease of the entropy in the rest of the non-equilibrium process may explain how the universe evolves to flatness. Interestingly, an inflationary dynamics in Bose-Einstein condensates (BEC) crossing a quantum critical point is recently observed^77^. Of course, the description of the inflationary dynamics of the early universe in terms of such a dynamical picture from the perspective of open quantum systems is only a conjecture. How to make this inflationary behavior of open quantum systems to become a more realistic cosmology model remains for further investigation.

**Figure 5:**
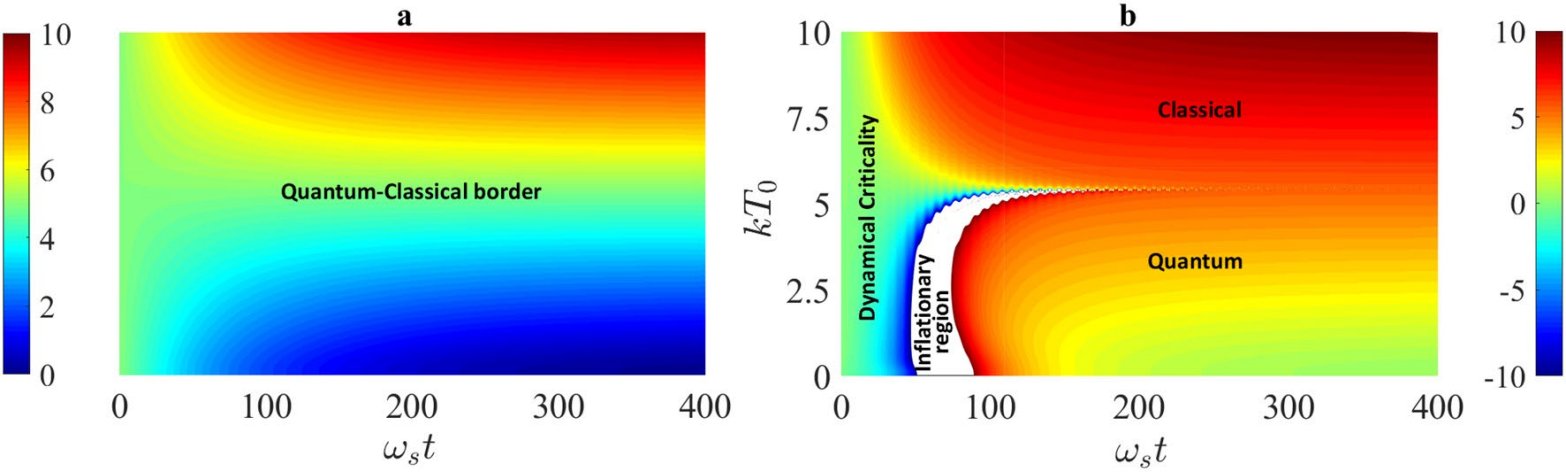
Dynamical quantum phase transition in quantum thermodynamics. Contour color-code plots of (**a**) the dynamical energy *E*(*t*) and (**b**) the dynamical temperature ${{\mathcal {T}}}(t)$ are presented as varying initial thermal energy $kT_0$ of the reservoir. The other parameters are taken as $\eta =0.01~\eta _c$, $\omega _c = 5 \omega _s$, and the system is considered to be in an initial state $|n_0\rangle =|5\rangle$. A dynamical quantum phase transition occurs when initial thermal energy $kT_0$ of the reservoir is very close to the initial system energy $E_0=5 \hbar \omega _s$.

Now if we change the initial state of the reservoir from vacuum to a thermal state with a low thermal energy, $kT_0 < E_0$, the dynamical entropy and the temperature of the system show the same behavior, except for the steady state of the system which becomes now a thermal state with temperature $T_0$ equilibrating to the reservoir, as shown by the red curves in Fig. 4. Thus, when we change the initial thermal energy ($kT_0$) of the reservoir from a lower value to a higher value crossing the system initial energy $E_0$ (or alternatively change the system initial energy from $E_0 < kT_0$ to $E_0> kT_0$ which is experimentally more feasible), we observe (see Fig. 5) a nontrivial dynamical quantum phase transition when the thermal energy $kT_0$ of the reservoir is very close to the initial system energy $E_0$. This quantum criticality provides indeed a dynamical border separating the classical regime from quantum regime, namely the classical regime quantitatively corresponds to a rather high temperature regime: $kT_0 > E_0$, while in the quantum regime: $kT_0 < E_0$, quantum thermodynamics can capture the occurrence of negative temperature and the decrease of entropy in the non-equilibrium process. To check whether this nontrivial quantum thermodynamical transition is generally true, we consider an another application, a two-level atomic system coupled to a bosonic reservoir in the next section.

### Application to two-level atomic systems

**Figure 6:**
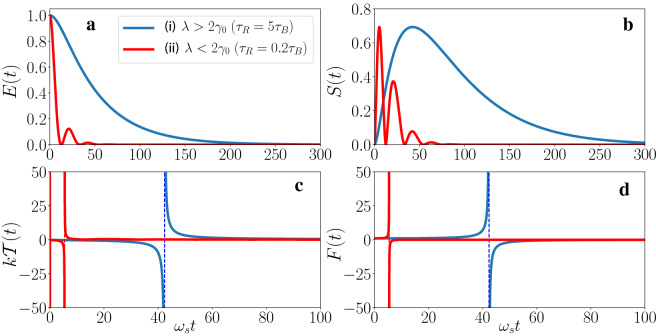
Non-equilibrium quantum thermodynamics of a two-level atomic system. The dynamics of non-equilibrium thermal parameters at two physically distinct regime (i) $\lambda > 2 \gamma _0$ and (ii) $\lambda < 2 \gamma _0$. We plot (**a**) average energy *E*(*t*) (**b**) entropy *S*(*t*) (**c**) non-equilibrium temperature ${{\mathcal {T}}}(t)$ and (**d**) free energy *F*(*t*) (in the unit of $\hbar \omega _s$ except for the entropy), where the blue curves represent weak coupling regime (i) with $\tau _R=5\tau _B$, and the red curves indicate the strong coupling regime (ii) with $\tau _R=0.2\tau _B$. The system is initially in the excited state, and the reservoir is initially in vacuum state.

To demonstrate the universality of the above finding in the simple cavity system described by the Fano-Anderson model, we investigate next the quantum thermodynamics of another widely studied open quantum system, namely a two-level atomic system (or a generalized spin-1/2 particle) interacting with a bosonic reservoir, to see how thermodynamics emerges from quantum dynamics of spontaneous decay process. The total Hamiltonian of the system plus reservoir is given by

20 $$\begin{aligned} H_{\mathrm{tot}} = \hbar \omega _s \sigma _{+} \sigma _{-} \!+\! \sum _k \hbar \omega _k b_k^{\dagger } b_k \!+\!\sum _k\! \hbar \! \left( g_k \sigma _{+} b_k + g_k^{*} \sigma _{-} b_k^{\dagger } \right) , \end{aligned}$$

which is also the basis of cavity QED in quantum optics that describes the matter-light interaction, where $\sigma _{+} = |1\rangle \langle 0|$ and $\sigma _{-} = |0 \rangle \langle 1|$ are the raising and lowering operators of the Pauli matrix with ground state $|0\rangle$, excited state $|1\rangle$, and transition frequency $\omega _s$. The reservoir Hamiltonian is described by a collection of infinite bosonic modes with the bosonic operators $b_k^{\dagger }$ and $b_k$, and $g_k$ is the coupling strength between the system and *k*-th mode of the reservoir. The general exact master equation for two-level system with the above Hamiltonian is still unknown. Here we consider that the system is prepared initially in the excited state $|1\rangle$ and the reservoir in a vacuum state. Then the time evolution of the reduced density matrix of the two-level system is given by the exact master equation^78,79^

21 $$\begin{aligned} \frac{d}{dt} \rho _S(t) =&- i \omega '_s(t,t_0) \left[ \sigma _{+} \sigma _{-} , \rho _S(t) \right] \nonumber \\&+ \gamma (t,t_0) \left[ 2 \sigma _{-} \rho _S(t) \sigma _{+} - \sigma _{+} \sigma _{-} \rho _S(t) - \rho _S(t) \sigma _{+} \sigma _{-} \right] , \end{aligned}$$

where the renormalized transition frequency $\omega '_s(t,t_0)= - \text {Im} \left( {\dot{u}(t,t_0)}/u(t,t_0) \right)$ and the dissipation coefficient $\gamma (t,t_0) = - \text {Re} \left( {\dot{u}(t,t_0)}/u(t,t_0) \right)$, which is the same as that given in the general exact master equation of Eq. (13). The Green function $u(t,t_0)$ satisfies also the same integro-differential equation as Eq. (14a)

22 $$\begin{aligned} \frac{d}{dt} u(t,t_0) + i\omega _s u(t,t_0) + \int _{t_0}^{t} d\tau g(t,\tau ) u(\tau ,t_0) =0, \end{aligned}$$

where the time non-local integral kernel $g(t,\tau )$ is related to the spectral density $J(\omega )$ of the reservoir as $g(t,\tau ) \!= \!\!\int \!\! d\omega J(\omega ) e^{-i\omega (t-\tau )}$. Here we consider a Lorentzian spectral density,

23 $$\begin{aligned} J(\omega ) = \frac{1}{2\pi }\frac{\gamma _0 \lambda ^2}{(\omega _0 - \omega )^2 + \lambda ^2}, \end{aligned}$$

for which the Green function $u(t,t_0)$ of Eq. (22) can easily be solved with the exact solution

24 $$\begin{aligned} u(t,t_0=0) = e^{(-i\omega _s -\frac{\lambda }{2})(t-t_0)} \Big ( \cosh \frac{\Gamma (t-t_0)}{2} + \frac{\lambda }{\Gamma } \sinh \frac{\Gamma (t-t_0)}{2} \Big ), \end{aligned}$$

where $\Gamma =\sqrt{\lambda ^2 - 2 \gamma _0 \lambda }$, which has no localized bound state because the spectral density covers the whole range of the frequency domain^21^. The spectral width of the reservoir $\lambda$ is connected to the reservoir correlation time $\tau _B$ by the relation $\tau _B \approx \lambda ^{-1}$. The relaxation time scale $\tau _R$ over which the state of the system changes is related to the coupling strength $\gamma _0$ by the relation $\tau _R \approx \gamma _{0}^{-1}$. The reduced density matrix $\rho _S(t)$ depicts the dissipative relaxation process of the system at far from equilibrium. Once we have the density matrix $\rho _S(t)$, the dynamical thermal parameters are determined by the relations (9–10). In Fig. 6, we show the dynamics of these thermal parameters, namely, the dynamical internal energy *E*(*t*), entropy *S*(*t*), temperature ${{\mathcal {T}}}(t)$ and the free energy *F*(*t*). We consider two different regime of the system-reservoir parameters: (i) $\lambda > 2 \gamma _0$, for which the reservoir correlation time is small compared to the relaxation time ($\tau _B < \tau _R$) of the system and the behavior of $u(t,t_0)$ shows a Markov exponential decay (ii) $\lambda < 2 \gamma _0$, in this regime the reservoir correlation time $\tau _B$ is large or comparable to the relaxation time scale $\tau _R$ where non-Markovian effects come into play^80^.

We investigate the non-equilibrium thermodynamics of the system in the above two physically distinct regimes. In the weak coupling case with $\tau _R=5\tau _B$, the energy *E*(*t*) shows a monotonous decay due to dissipation, as shown in Fig. 6a (the blue curve). On the other hand, Fig. 6b (the blue curve) shows that the entropy *S*(*t*) increases due to the contact of the system to the reservoir, and attains a maximum value at an intermediate time, henceforth the entropy starts decreasing and eventually approaches to zero. This happens because the system is initially in a pure state (zero entropy), then the entropy increases during the non-equilibrium process and reach the maximum corresponding to maximum mixing state of the system, finally the entropy goes to zero again when the system relaxes into the vacuum state (pure state) in order to equilibrate with the reservoir. The decrease in energy with increasing entropy results in a negative dynamical temperature of the system, as shown in Fig. 6c. The dynamical temperature ${{\mathcal {T}}}(t)$ decreased to negative infinity as the entropy approaches to the maximum, it then jumps from negative infinite temperature to positive infinite temperature at the maximum entropy point, eventually the system reaches to zero temperature at the steady state as shown in Fig. 6c, the exactly same as what we find in the cavity system in the weak-coupling quantum regime.

The physical origin of negative dynamical temperature for this two-level atomic system and the associated inflationary behavior are also the same as that in the cavity system: The free energy increases instead of decreasing (see Fig. 6d). Hence, for this two-level system, the second law of thermodynamics also *cannot* be maintained when the system evolves from a pure state to a mixed state and then back to a pure state in the whole spontaneous decay processes. Nevertheless, the entropy and the temperature both dynamically approach to zero in the steady-state limit, as a manifestation of the third law of thermodynamics in equilibrium state in the quantum regime. In the strong coupling case with $\tau _R = 0.2 \tau _B$ (see the red curves of Fig. 6), we show the non-equilibrium thermodynamics in the regime $\lambda < 2 \gamma _0$. In this regime, the average energy *E*(*t*) initially decreases, then it shows a small oscillation, demonstrating a backflow of energy (red curve of Fig. 6a) from the system into the reservoir, as a non-Markovian memory effect. We see a similar dynamical behavior in the entropy *S*(*t*) under this strong coupling, namely, the entropy increases in the short time, then decreases and oscillates in later time (red curve of Fig. 6b). The dynamical temperature and free energy in the strong coupling regime (red curves of Fig. 6c, d) also show similar behaviors except a small shift in the time-scale. The dynamical transition associated with the inflationary dynamics is the same as we found in the single cavity system.

The concept of negative temperatures has indeed been discussed in the literature^81,82^ which demands the system to be in the equilibrium and also to have a finite spectrum of energy states, bounded both from the top and from the bottom. Such negative equilibrium temperature has been realized in localized spin systems^83–85^, where the discrete finite spectrum naturally provides both lower and upper energy bounds. A similar situation is also found recently where a cloud of potassium atoms is tuned to negative temperatures via quantum phase transition^86^. However, the physical origin or the condition under which the negative temperature phenomenon occurs in our cases is quite distinct from the previous studies^81–86^. In previous studies^81–86^, it was emphasized that in thermal equilibrium, the probability for a particle to occupy a state with energy $$E_n$$ is proportional to the Boltzmann factor $$\exp (- E_n/kT)$$. For negative temperatures, the Boltzmann factor increases exponentially with increasing $$E_n$$ and the high-energy states are then occupied more easily than the low-energy states. Contrary to that, the emergence of negative temperature here we see is dynamical, and it has a clear physical origin in the non-equilibrium process that the entropy increases and then decreases as the system starts from a pure state, evolves into a maximum mixed state and then returns back to a pure state (or a less-maximized mixed state) dominated by the spontaneous emissions when the reservoir is initially in vacuum state (or an initial state has a very low thermal energy). As long as one can prepare the system in a pure state with energy $$E_0$$ and the reservoir in a thermal state with initial thermal energy $$kT_0$$ such that $$ kT_0 < E_0$$ (the quantum regime), the occurrence of a negative dynamical temperature and the associated inflationary behavior are universal behaviors in the thermalization processes of open quantum systems.

## Conclusions and outlook

Thermodynamics is built with the hypothesis of equilibrium, where dynamics in time is absent and all thermodynamic parameters are time-independent. In this paper, we develop a non-equilibrium theory for quantum thermodynamics validating even for single particle quantum systems coupled with reservoirs far from equilibrium. We generalize the concept of temperature and other thermodynamic parameters to non-equilibrium regime that depends on the details of quantum states of the system and their time evolution. We show that the theory unravels the intimate connection between the laws of thermodynamics and their quantum origins. We explicitly and exactly solved the dynamical evolution of two single quantum systems interacting with a thermal reservoir described by the Fano-Anderson model and multimode Jaynes-Cummings (JC) model, respectively. These are the two most general examples that can be solved unambiguously in open quantum systems. We demonstrated the emerging thermodynamics of the system through the time evolution of various thermodynamic parameters, namely the dynamical energy, entropy, temperature, free energy, in the weak and intermediate system-reservoir coupling regimes under the situation of the system far from equilibrium. We showed how the system dynamically approaches to a thermal equilibrium state asymptotically in the steady-state limit. In the strong system-reservoir coupling regime if there exist localized bound states^21^, a revival dynamics in the thermodynamic parameters is observed, indicating back-reactions between the system and the environment as a long-time non-Markovian memory effect^70^. Consequently, equilibrium thermodynamics is unreachable when the system has localized mode or localized bound state, as noticed early by Anderson^49^ and justified recently by one of us^36^. However, if the spectral density is continuous and covers the whole energy range so that no localized bound state can be generated by the couplings between the system and reservoirs, then thermalization is always reachable.

The other remarkable findings in the quantum thermodynamics appear when the reservoir thermal energy $kT_0$ being less than the system initial energy, which is defined as the quantum regime. Under this situation, we observe a dramatic change in the non-equilibrium thermodynamic behavior of the system. This dramatic change induces an inflationary dynamics which may provide a simple picture for the origin of big bang and universe inflation. The ending steady state in this situation manifests the third law of thermodynamics in the deep quantum realm. We also observed a nontrivial dynamical quantum phase transition when the thermal energy of the reservoir is very close to the system initial energy. The corresponding dynamical criticality provides a border separating quantum and classical regimes. This dynamical quantum phase transition is manifested through non-equilibrium quantum processes that cannot be captured by equilibrium thermodynamics itself.

These new findings for quantum thermodynamics could be examined through BEC experiments or cavity QED experiments. More specifically, one can experimentally explore the photon dynamics or boson dynamics via quantum non-demolition measurement^87,88^ to justify these findings. For example, by preparing the cavity in a Fock state, and sending sequences of circular Rydberg atoms through the photonic cavity in different environment, which carry the cavity state information without destroying the cavity photon state. The cavity photon state can be measured using an experimental setup similar to that given in Refs.^87,89^. Another way to observe the nontrivial quantum thermodynamics we obtained is to measure the energy loss and the state evolution of a two-level atom in cavity QED system. It may be also possible to use the BEC systems^77^ to find these new features. On the other hand, it is also straightforward to apply this quantum thermodynamics theory to electronic systems in nanostructures where the exact master equation with quantum transport has been developed^21,90^.
